# Behavior of a Person-Following Robot in Pedestrian Environments: Laboratory Experimentation and Simulation

**DOI:** 10.1177/03611981251332248

**Published:** 2025-04-30

**Authors:** Ruowei Li, Farah Ghizzawi, Tho V. Le, Matthew J. Roorda

**Affiliations:** 1 Department of Civil & Mineral Engineering, University of Toronto, Toronto, ON, Canada; 2 School of Engineering Technology, Purdue University, West Lafayette, IN, USA

**Keywords:** city logistics and last-mile strategies, human factors, pedestrians, simulation delivery robot, robot-pedestrian interaction

## Abstract

As e-commerce continues to grow, the demand for fast and efficient last-mile delivery is increasing. Person-following robots is a solution that is now beginning to be deployed to assist foot couriers in performing last-mile delivery tasks in public areas, such as underground pedestrian walkways and malls. Although they have proved their capacity to operate successfully in uncrowded and highly regulated spaces like warehouses, it is still uncertain whether their performance would be satisfactory in crowded, unstructured, and complex environments. This study proposes a simulation approach for evaluating the performance of a commercial person-following delivery robot in dynamic pedestrian environments. Laboratory experiments were conducted to understand the robot’s operating characteristics. Based on empirical observation, a computer simulation model of the robot was developed in a pedestrian simulator, which was calibrated and validated with experimental data. This model was expanded to include pedestrian crowds such that the performance of the robot was evaluated under various crowding scenarios. Research limitations and recommendations for future work are identified.

Online business-to-consumer transactions have undergone rapid growth in recent years. The U.S. Department of Commerce (*1*) has forecasted a 12.5% increase in retail e-commerce sales worldwide by 2026. Disruptions caused by the COVID-19 pandemic have not only affected individual consumers but also businesses. Small business-to-business firms in the UK and Brazil, for example, saw large gains in online income compared with pre-COVID numbers (*1*).

The rise of e-commerce is accompanied by increased customer expectations for fast, affordable, and reliable delivery. Over the years, diesel-engine vehicles have dominated last-mile deliveries and have contributed to traffic congestion as well as noise and air pollution in urban environments. Delivery delays are also common, resulting in unnecessary shipment costs for shippers and receivers. These challenges have motivated innovative concepts of delivery in the freight industry. The use of electric autonomous delivery robots, as a potential solution, has been widely discussed in recent years.

This paper investigates the operation of a person-following delivery robot, EffiBOT, and introduces a framework to evaluate its performance in various pedestrian environments through computer simulation. This framework encompasses laboratory experiments with the actual robot, the development, calibration, and validation of the simulated robot, and its application in a virtual pedestrian environment. Ultimately, this simulation study aims to provide quantitative evidence to assist government agencies and logistics companies in making informed decisions about the effective deployment of this technology in public spaces.

## Autonomous Vehicles in Freight Industry

In the logistics industry, prospective applications of autonomous vehicles (AVs) include indoor warehouse, and controlled outdoor, long-haul, and last-mile delivery operations. AVs in controlled (private) outdoor environments such as intermodal terminals and logistics yards is one of the most established uses of AVs in logistics. These vehicles are referred to as “automated guided vehicles” or “autonomous mobile robots,” and they usually support the (un)loading processes, the (un)stacking processes, the gate deliveries, and pickups. Similar technologies are also utilized in warehouses and distribution centers, performing tasks such as order picking. The deployment of these vehicles increases efficiency, reduces the physical burden on workers, and frees up personnel to concentrate on higher-value duties, such as quality control (*2*).

To date, applications of AVs in public environments are limited. One promising application is automated truck platooning for long-haul freight transport. Pilot studies have been carried out around the world, with results indicating that truck platooning reduces energy consumption and emissions, reduces human errors, and increases safety and productivity (*3*, *4*).

The use of AVs for the last mile is also in its early stages. According to industry estimates, last-mile delivery is costly and time-consuming (*5*), encouraging courier companies to seek solutions such as AVs to facilitate last-mile delivery. These AVs are generally referred to as “autonomous delivery vehicles.” Two robotic platforms are now being tested: unmanned aerial vehicles (UAVs) and autonomous delivery robots (ADRs).

UAVs, also known as drones, are mostly used to deliver retail products, food, and medical supplies in last-mile applications. They have the potential to shorten delivery times and reduce environmental side effects. ADRs are another type of technology, with the three variations being on-road autonomous delivery robots (RADRs), sidewalk autonomous delivery robots (SADRs), and indoor autonomous delivery robots (IADRs). RADRs are compact versions of self-driving cars intended for cargo delivery and usually travel on streets. In contrast, SADRs are generally box-sized robots that deliver food or packages to customers and are designed to roll on sidewalks at walking speeds. IADRs are generally box-to-human-sized robots that perform delivery or other service tasks in an indoor environment.

## Application of Autonomous Delivery Robots in Last-Mile Delivery

SADRs are being tested through pilot studies globally by both small and large firms. Some examples are Amazon Scout, Kiwi, and Starship (*6*). The delivery items range from groceries to medical supplies and food. They also operate in a variety of environments such as suburban neighborhoods and university campuses.

**Figure 1.**
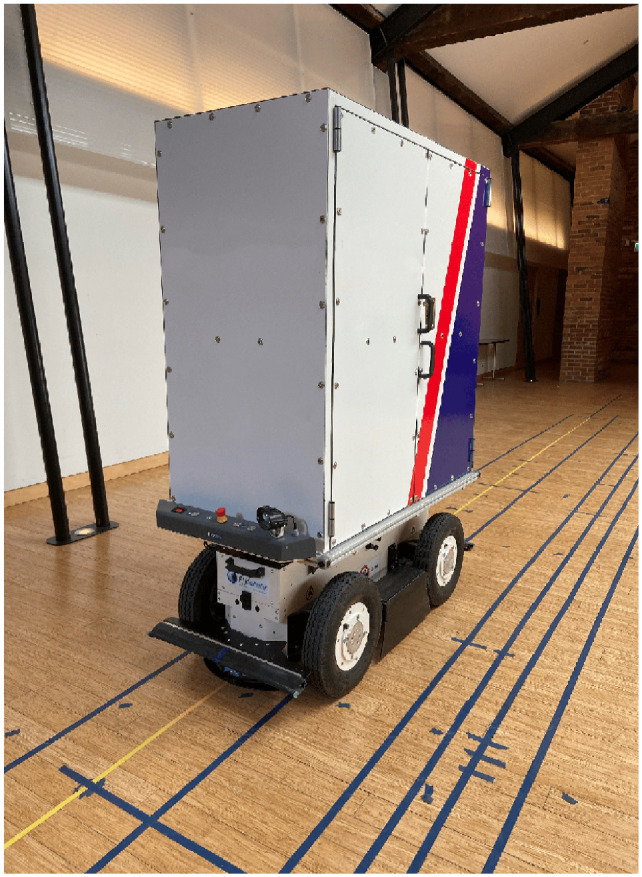
EffiBOT.

Person-following robots are a type of robot technology used in both sidewalk and indoor environments. To date, the primary use of person-following robots in logistics is in warehouses for collaborative automatic order picking. These robots detect and follow the picking operators autonomously using camera and/or LiDAR sensors, enabling the pickers to work without undue physical exertion. The application of person-following robots in last-mile delivery, however, is still relatively new. In 2017, a pilot study was conducted by the German postal service to test robot-assisted mail delivery in a small German town using PostBOT (*7*). Another pilot study that is underway of a person-following delivery robot is EffiBOT (Figure 1), being tested by a Canadian express package delivery company. It is a four-wheel-drive intelligent handling robot of dimensions 1.3- (length) × 0.7- (width) × 1.8 m (height) and a weight of 130 kg. Instead of replacing the human courier, these robotic parcel carriers are used to help staff with their physically challenging work, as they carry all the heavy mail items for the courier and follow them closely while they are on their route for delivery.

## Pedestrian Factors in Last-Mile Delivery Using Autonomous Delivery Robots

The interaction between robots and pedestrians is a frequent talking point in public discourse around autonomous robots in public spaces. Since human motion is dynamic and sometimes unpredictable, conflicts are likely as more ADRs enter the public realm. Citizens have raised concerns about how sidewalks should be used and shared between automated robots and pedestrians (*8*). It is believed the lack of quantitative evidence on robot performance in pedestrian areas is preventing cities from embracing a wider adoption of SADRs (*9*, *10*).

As of today, the literature about the performance of mobile robots within pedestrian environments and in the context of last-mile delivery is still limited. Most studies covering this issue focus on the technology, for example, the use of different types of sensors on service robots as well as human-aware navigation and collision-avoidance algorithms (*11*, *12*). On the other hand, logistics literature that studies the impact of ADRs in the context of last-mile delivery mostly focuses on freight efficiency and the environmental, economic, and legal implications, while overlooking the presence of pedestrians and the impact of microscopic human–robot interaction (HRI) in urban settings (*13*, *14*). An attempt was made to quantitatively assess delivery robots’ performance and social acceptance in a pedestrian environment on a university campus (*15*). However, the scale of the tests was relatively small, and the data were collected from manual observations, which may have affected the precision of the results. Furthermore, the data collected by individual corporations from the pilots are likely confidential and inaccessible to policy makers for making evidence-based decisions.

The lack of understanding of how ADRs perform in dynamic pedestrian environments motivates this research. This paper presents a simulation-based method for assessing the performance of a robot in dynamic pedestrian environments.

## Paper Organization

The remainder of this paper is structured as follows. The next section reviews literature on SADRs, IADRs, person-following robots, and pedestrian simulation models. Section 3 describes the laboratory experiments conducted to understand the person-following and obstacle-avoidance behavior of EffiBOT. Section 4 covers the implementation of the robot model, followed by Section 5, which focuses on the calibration and validation of the model. Section 6 discusses the performance evaluation of the person-following robot under various crowding scenarios using an agent-based simulation model. The last section concludes the research and discusses the opportunities for future work.

## Literature Review

The goal of this study is to understand the performance of a person-following robot in pedestrian environments within the context of last-mile delivery. Since the robot is not designed to operate on roads, this review focuses on studies related to SADRs and IADRs. Pedestrian simulation algorithms and tools are reviewed to assess the state-of-the-art simulation methods available for creating virtual pedestrian environments. Additionally, person-following algorithms and HRI studies are examined to inform the development of the robot simulation model.

### Sidewalk Autonomous Delivery Robots for Last-Mile Delivery

Literature covering the application of SADRs in last-mile logistics ranges from describing the new delivery mode (conceptual), examining the technology itself (technical), to analyzing the efficiency this mode can potentially bring (optimization).

Conceptual papers discuss the potential of SADR technology, its prospective applications in last-mile delivery, and the legal and societal implications of adopting this technology (*16 [Bibr bibr17-03611981251332248]*–*18*). Technical papers focus on delivery robots’ mechanical, electrical, and software system requirements (*12*, *19*). Several research papers are devoted to solving vehicle routing and scheduling problems, aiming to analyze the efficiency and optimize various autonomous delivery systems (*14*, *20 [Bibr bibr21-03611981251332248]*–*22*).

### Indoor Autonomous Delivery Robots for Last-Mile Delivery

In the past few years, several indoor delivery robots have been developed, some of which have already been commercialized. These professional service robots are operated in various indoor environments such as restaurants (*23*), hospitals (*24*), and hotels (*25*).

Since global positioning systems are inoperable indoors, several studies focus on indoor localization and mapping. Information from multiple sensors is collected and fused to construct indoor maps for robots to navigate through (*23*, *26*, *27*). Some researchers explored the multifunctionality of indoor service robots. Modular hardware components were implemented so that the robots could perform not only delivery tasks, but also cleaning, guiding, and patrol tasks (*28*).

Other literature discusses route planning and optimization (*29*, *30*). For example, Kim and Jung proposed a heuristic approach for a delivery robot routing problem inside a multistory building, while considering the characteristics of robot movement and the indoor environment (*31*).

The COVID-19 pandemic increased interest in contactless interaction. Choi et al. designed an indoor beverage delivery robot that can be controlled by users through verbal communication, in the hope of maintaining social distance and improving health and safety for employees and customers (*32*).

### Pedestrian Models and Simulation Tools

Numerous developments have been made in the field of pedestrian simulation. Owing to limitations in computing power, several early pedestrian models used a macroscopic approach. Examples include simple regression models (*33*), network models (*34*), and fluid-dynamic models (*35*). With recent increases in computational power, microscopic models have been developed. These models focus on the behavior and choices of individual pedestrians and their interactions with other pedestrians in a crowd. Helbing and Molnár proposed the original social force model (SFM), which assumed that pedestrians feel internal motives for a certain action when walking, depending on the “social forces” they encounter in their surroundings (*36*). Helbing et al. (*37*) and Helbing and Johansson (*38*) extended the model by introducing panic situations and the elliptical interaction force formulation. Another group of pedestrian models is based on the concept of cellular automata (CA) and operates in discretized space. Literature on CA models focuses on two typical scenarios: bidirectional walking flow (*39*, *40*) and emergency evacuation (*41*). Inspired by both SFMs and CA models, the optimal steps model (*42*) was developed to combine the versatility of working in continuous space with the ease of working with discrete motions, unconstrained by a cellular grid. Aside from the aforementioned agent-based models, researchers have also proposed utility-based models (*43*, *44*). These models rely on utility theory, whereby individuals make decisions based on the perceived benefits or costs of various choices. Utility-based models are often used in situations where pedestrian decisions involve tradeoffs, such as route selection, speed, or interaction preferences. However, macroscopic models, CA models, and utility models are less relevant to this study, as this study focuses on modeling a robot’s behavior when it interacts with static and dynamic objects (i.e., pedestrians) in its surrounding environment.

Pedestrian simulation refers to the representation of crowd dynamics using computer algorithms. Today, various commercial pedestrian simulators, such as MassMotion (*45*) and PTV Viswalk (*46*), are available and convenient to use. These tools integrate one or more pedestrian models into the virtual simulation environment and allow users to adjust a few model parameters and fine-tune their simulations to fit certain requirements. Research-sourced pedestrian simulators, such as Menge (*47*), were also developed. Although less user-friendly compared with its commercial counterparts, Menge’s modular framework and plug-in architecture allow it to be dynamically configured with much flexibility, which greatly facilitates comparisons between algorithms.

### Human and Robot Interaction and Social Awareness

An increasing number of autonomous robots have been introduced into the public realm and participated in people’s daily lives. Whether these robots can navigate through dynamic human environments in a safe and socially compliant manner plays an essential role in facilitating human–robot coexistence and improving public acceptance of such robots. In this context, HRI and socially aware navigation are of growing interest.

Socially aware navigation refers to robots’ ability to plan a collision-free path toward a goal location without causing discomfort to nearby humans (*48*). Conventional mobile robot navigation approaches usually consider humans as regular obstacles and apply classical global and local planning methods for optimal and collision-free path planning, however, they often overlook the aspect of psychological safety. Research efforts have been made to address the drawbacks of conventional navigation approaches in recent years. The new methods include proxemics-theory-based cost maps (*49*), machine learning (*50*), and social-force-inspired algorithms (*11*, *51*).

### Person-Following Algorithms

Collaborative robots are also capable of serving a wide range of purposes such as companionship, helping with daily activities, and providing physical and emotional support. A common characteristic of these robots is that they work collaboratively with a human to complete a common task that calls for the robot to follow the human. To follow a person, the robot must first perceive the relative position of the target person in its operating environment and then navigate toward the person safely and naturally (*52*).

Numerous methods have been proposed for human detection and tracking, which can be classified into three categories: feature-based tracking, feature-based learning, and feature or representation learning. Once the designated person’s position and heading are estimated, this information is used by the path planner to find the optimal trajectory toward them while also recognizing the static obstacles and other people in the environment. The common approaches include direction-following, path-following, and relative-following.

### Pedestrian Simulation Tools for Robot Navigation

Computer simulation is suitable for studying autonomous robot performance in dynamic pedestrian environments. Currently, specialized pedestrian simulation tools such as MassMotion (*45*) and Viswalk (*46*) do not simulate robots, and most robotics simulators do not incorporate pedestrian movements. However, several simulation approaches can be found in the literature. MengeROS (*53*) is an open source two-dimensional (2D) simulator that integrates the pedestrian simulator Menge with Robot Operating System, which can simulate robots in various crowd scenarios. Ferrer et al. developed simulations for robots and pedestrians, but the simulation platform was not described (*54*, *55*). Kirkland and Maciejewski studied the impact of introducing autonomous robots into crowds using simulation approaches (*56*). The results indicate that robots can modify crowd dynamics and improve pedestrian flow. Additionally, researchers have developed simulation tools using gaming engines (*51*).

To the best of the authors’ knowledge, there is currently no simulation reported in the literature that simultaneously incorporates pedestrians and person-following robots. This paper proposes a framework to simulate and calibrate a commercial person-following delivery robot. The resulting model could then be used to examine the performance of this type of robot in virtual pedestrian environments.

## Laboratory Experimentation

Empirical experiments are a crucial component in understanding HRI. Controlled experimental areas are often constructed to conduct tests, where human behaviors are tracked by motion trackers to generate trajectories (*43*, *44*, *57*). Perceived safety or comfort levels are commonly assessed through participant questionnaires with Likert scales (*57*, *58*). The behavior or positioning of the robot is almost always controlled by the robot platform (*43*, *44*, *59*). However, in this study, EffiBOT followed its leader and interacted with other objects with its inherent parameter settings, as the robot platform’s I/O (input/ouput) was inaccessible to the researchers. The behavior of EffiBOT could only be observed and tracked using a third-party device.

The laboratory experiments were conducted to observe EffiBOT’s person-following behavior and its reactions to obstacles and pedestrians. These experiments provided insights into the logic governing the robot’s movements in various working scenarios and informed the selection of the robot’s simulation algorithm. The trajectory data obtained from these experiments were used to calibrate the robot model parameters and served as the ground truth for model validation. The simulated robot trajectories were then compared with the observed trajectories using relative distance error as a metric.

### Experiment Setup and Data Collection Method

The experiments covered scenarios that can frequently occur when robots and humans navigate the same environment, as well as some extreme cases. More specifically, they were classified according to the level of complexity of the interaction between the robot and its surrounding environment. The experiments included the robot, the operator/leader, and static obstacles and/or dynamic obstacles. Static obstacles were represented by cardboard boxes, and the dynamic obstacle was a researcher acting as a “pedestrian.” Within each category, a set of experiments was designed, yielding 12 types of experiment, as shown in Table 1. These 12 types of experiment were further differentiated by the operator’s walking speed, the distance from the obstacles, and different routes, resulting in 45 subexperiments.

**Table 1.** Experimental Categories

Categories		Experiments	
A	Robot + Operator	1	Straight line following test
		2	Circular line following test
B	Robot + Operator + Static obstacles	3	Corner test
		4	Static obstacle test (passing)
		5	Static obstacle test (avoidance)
		6	Obstacle map
C	Robot + Operator + Pedestrian	7	Pedestrian interference test (surpass)
		8	Pedestrian interference test (encounter)
		9	“Lose target” test (crossing)
		10	“Lose target” test (takeover)
D	Robot + Operator + Static obstacles + Pedestrian	11	Static & dynamic obstacles test (surpass)
		12	Static & dynamic obstacles test (encounter)

**Figure 2.**
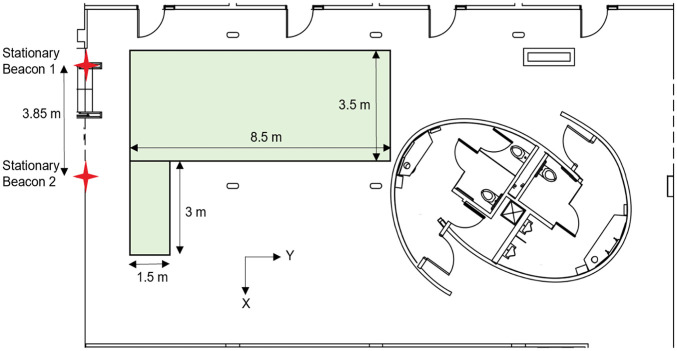
Stationary beacon locations and experiment area.

All experiments were conducted on the 4th floor of the Lassonde Mining Building at the University of Toronto. An ultrasound-based indoor-localization system by Marvelmind was deployed to record time-stamped location information for the walkers and the robot. The central controller of the navigation system was connected to a laptop during the experiments. Two stationary beacons were placed on the walls 3 m above the floor and 3.85 m apart to generate a 2D map of the experiment area, as shown in Figure 2. The accuracy of the measurement primarily depends on room temperature, with the absolute distance error being 0.17%/°C of the actual distance between the beacons (*60*). The accuracy was deemed reasonable based on experimental observations. A mobile beacon transmitting ultrasound signals was placed top center of the robot to track its location. To obtain the coordinates of the robot operator and the pedestrian, they wore hard hats with mobile beacons attached to them. The time-stamped coordinates of each beacon were extracted, and velocity profiles were derived by differentiating the displacement.

### Experimental Results

#### Experiment Category A

Category A studied the robot’s basic following behavior under the simplest working condition: the operator walked along a straight line or a circular line with no obstacles present near the route. The results showed that the highest linear speed the robot could reach was 1.44 m/s (i.e., 5.2 km/h). As the operator’s speed increased, the robot’s following distance also increased, and it took the robot longer to accelerate and catch up with the operator. Additionally, the robot would keep a safety distance of approximately 0.35 m from the operator when it came to a full stop. The circle test showed that the robot could turn as fast as 45°/s and would closely follow the operator’s route if the operator walked slower than 4 km/h. It was found that large-angle steering may compromise its following speed.

#### Experiment Category B

Category B incorporated static obstacles in the experimental setup to understand EffiBOT’s behavior when encountering obstacles such as walls or objects. Four scenarios were tested: the operator (1) turned left at a corner; (2) passed by a single cardboard box; (3) moved around a single cardboard box; (4) maneuvered through an obstacle map.

**Figure 3.**
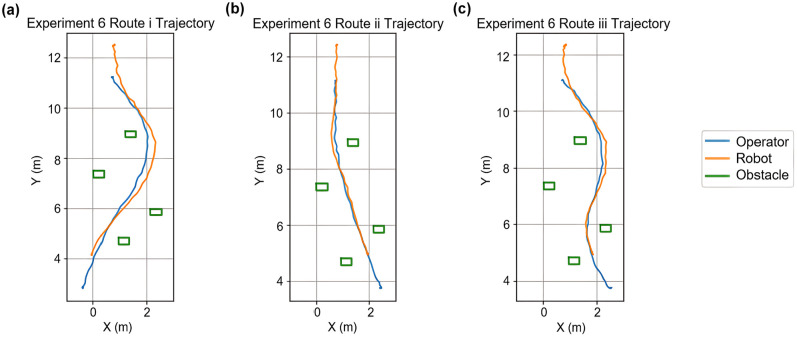
(*a*) Experiment 6i trajectory, (*b*) Experiment 6ii trajectory, and (*c*) Experiment 6iii trajectory.

When turning at a 90° corner, being in close vicinity to the wall, and being led by a fast-moving operator both negatively affected the robot’s ability to follow. The robot would stop at the corner when it followed a route that was 0.60 m to the walls and the operator walked at a speed greater than or equal to 4 km/h. When the distance was 0.75 m, the robot would follow along to the end if the operator walked at approximately 4 km/h, but would stop at the corner if the operator increased their speed to around 6 km/h. When passing by a single cardboard box or traversing through an obstacle map comprised of four cardboard boxes, the robot was able to safely maneuver around them, even though it had to make detours when the operator walked too close to these obstacles (Figure 3). The experiments showed that the minimum distance the robot would keep from the static obstacles was approximately 0.13 m from its side.

#### Experiment Category C

In Category C, dynamic obstacles (i.e., pedestrians) were introduced. Experiments 7 to 10 were designed to test how the robot would react to a pedestrian as the pedestrian passed by it, walked toward it, or tried to confuse the robot by going between it and its operator.

It was found that a single pedestrian walking in parallel with the robot had little impact on its speed or trajectory, despite the distance between the two. The robot would steer slightly away from the pedestrian and keep a safe distance of at least 0.04 m from its side to the pedestrian. When the pedestrian caused more disruption by crossing between the robot and its operator or trying to “replace” the operator by walking next to the operator and then moving in a different direction, the robot was not confused but followed the operator to the end. When the pedestrian cut through the robot and the operator twice during one run, the distances between the robot’s front bumper to the pedestrian were 0.38 and 0.62 m, and the robot’s speed then dropped to approximately 0.1 m/s, although the pedestrian had already moved to the side during the robot’s deceleration.

#### Experiment Category D

Category D simulated a rather complex scenario with a static and a dynamic obstacle on both sides of the robot’s planned route. The purpose was to observe how EffiBOT would react in a confined space when close to static and dynamic obstacles. The operator would walk along a straight line at an average walking speed, surpass a static cardboard box along the way, while a pedestrian would either catch up from behind or walk toward it.

**Figure 4.**
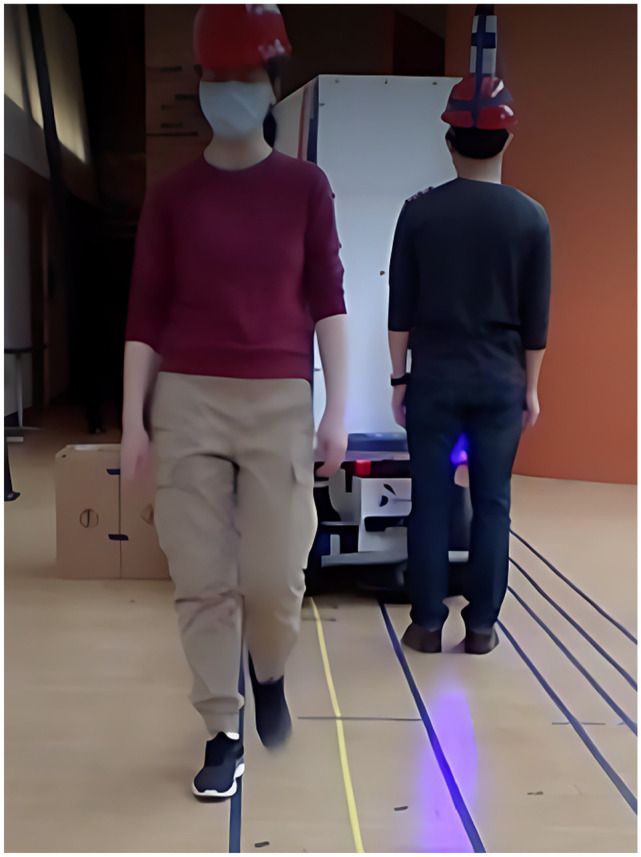
Experiment 12, the robot blocking the pedestrian’s route.

When the distance from the obstacle’s edge to the planned route was greater than or equal to 0.50 m, the robot would generally stay close to its route, no matter how close its distance to the pedestrian. Compared with the results from Experiments 4, 7, and 8, it can be inferred that being confined between two obstacles restricted the robot’s maneuverability. In Experiment 11, the minimum distance the robot kept between the static obstacle and its side was 0.11 m, which was consistent with the finding in Experiment 6. When the operator walked along a route 0.25 m away from the obstacle and 0.60 m away from the pedestrian who walked in parallel in the opposite direction, the robot would block the pedestrian’s route as it tried to maneuver around the static obstacle (Figure 4). If the pedestrian stood still where this collision was about to occur, it could take the robot up to 8 s to find its way between the obstacles, and it ceased to follow completely in one of the three trials. In all trials, the robot started decelerating when its bumper was approximately 0.45 m away from the pedestrian.

#### Summary of Experimental Results

In general, the experimental results showed that the robot tracked the operator more precisely when the operator moved at a slow to medium walking speed (i.e., ≤ 4 km/h). It struggled to keep up when the operator made sharp turns, possibly because of its kinematics design, which makes it less agile than smaller omnidirectional delivery robots. The experimental results also suggested that the robot was more aware of the obstacles ahead of it than either side of it. It would keep a larger safe distance from the obstacles in its moving direction, and a shorter safe distance from the obstacles either side. Furthermore, the robot was able to recognize the correct leader in cases of pedestrian disturbance and keep a safe distance from all static and dynamic obstacles around it. When steering away from obstacles was not feasible, it would decelerate to avoid collisions. In the worst-case scenario, it would stop following and require human intervention. Thus, to ensure smooth operation in practice, it is recommended that the courier maintains a low to medium walking speed, avoids making sharp turns, and follows routes that are distant from any obstacles.

## Robot Simulation Algorithm Implementation

The robot’s simulation model was built in MassMotion (*45*) using the MassMotion Software Development Kit (SDK). MassMotion is one of the most prevalent pedestrian simulation software tools that models agent behavior using the social-force concept. The MassMotion SDK allows for the customization of individual agent behaviors. Since it is believed that applying calibrated force-based pedestrian models to a robot for reproducing humanlike collision-avoidance behavior is not only computationally efficient but also more stable to environmental changes than traditional approaches (*43*), and having the simulated robot and pedestrians interact under similar force-based rules is desirable, a modified SFM was developed for the robot in the hope of replicating the robot behavior observed during real-life experiments.

**Figure 5.**
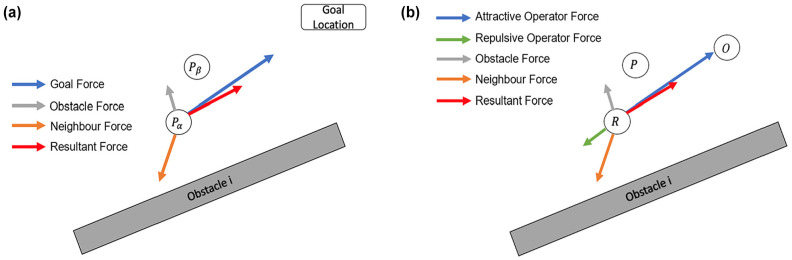
(*a*) Social forces exerted on a pedestrian, $P_{\alpha }$ , and (*b*) social forces exerted on a person-following robot, *R*.

The SFM (*38*) is an agent-based model, which states that the motion of each individual in a crowd is subjected to the superposition of three forms of “virtual force”: an attractive force to their intended goal location, a repulsive force from other pedestrians, and a repulsive force from static obstacles (Figure 5*a*). In this research, the underlying logic that dictates the robot’s person-following and obstacle-avoidance behavior is approximated using a modified SFM. Although the task to avoid potential collisions with other pedestrians and static obstacles can be the same for a person-following robot, this proposed model acknowledges that instead of a stationary goal location, a person-following robot is attracted to a moving object (i.e., the operator), and it is also repelled by the operator. To summarize, a person-following robot is subjected to forces from four sources: attractive and repulsive forces coming from its operator, repulsive forces from surrounding pedestrians, and repulsive forces from nearby obstacles (Figure 5*b*).

Equation 1 summarizes the resultant force. The first term describes the attraction from the operator to the robot. The goal direction is calculated as the difference between the current operator position, ${\vec{r}}_{O}$ , and the current robot position, ${\vec{r}}_{R}$ , and is multiplied by the current operator speed, $v_{O}$ , to represent the desired robot velocity. This quantity is subtracted by the robot’s current velocity and then divided by $\tau_{r}$ , the relaxation term that smooths the acceleration of the robot.

(1) $$\begin{array}{cc} {\vec{f}}_{R}\left(t\right) & =\frac{1}{\tau_{R}}\left(v_{O}\times \left({\vec{r}}_{O}-{\vec{r}}_{R}\right)-{\vec{v}}_{R}\right)+w(\varphi_{\mathrm{RO}}\left(t\right)){\vec{f}}_{\mathrm{RO}}\left(t\right)\\ & +\sum_{P}w\left(\varphi_{\mathrm{RP}}\left(t\right)\right){\vec{f}}_{\mathrm{RP}}\left(t\right)+\sum_{i}{\vec{f}}_{\mathrm{Ri}}(t) \end{array}$$

The second and third terms ${\vec{f}}_{\mathrm{RO}}\left(t\right)$ and ${\vec{f}}_{\mathrm{RP}}\left(t\right)$ are the repulsive forces from dynamic obstacles (i.e., the operator, O, and nearby pedestrians, P). These forces are formulated similarly, with different parameter values used for the operator and other pedestrians, respectively. The repulsive force has an elliptic formulation and is calculated as in Equation 2, with $b_{\mathrm{RP}}$ , ${\vec{d}}_{\mathrm{RP}}$ , and ${\vec{y}}_{\mathrm{RP}}$ defined by Equations 3, 4, and 5. According to this formulation, the robot, as an agent in simulation, would feel more repulsion from individuals in its moving direction than the ones on the side, implying that when there is a chance of a head-on collision between two agents, the agent has a strong inclination to decelerate. The higher the confronting speed, the greater the magnitude of the repulsion.

(2) $$\begin{array}{cc} {\vec{f}}_{\mathrm{RP}}\left(t\right)= & A_{P}e^{\frac{-b_{\mathrm{RP}}}{B_{P}}}\times \frac{\left\|{\vec{d}}_{\mathrm{RP}}\|+\|{\vec{d}}_{\mathrm{RP}}-{\vec{y}}_{\mathrm{RP}}\right\|}{2b_{\mathrm{RP}}}\times \\ & \frac{1}{2}\left(\frac{{\vec{d}}_{\mathrm{RP}}}{\left\|{\vec{d}}_{\mathrm{RP}}\right\|}+\frac{{\vec{d}}_{\mathrm{RP}}-{\vec{y}}_{\mathrm{RP}}}{\left\|{\vec{d}}_{\mathrm{RP}}-{\vec{y}}_{\mathrm{RP}}\right\|}\right) \end{array}$$

(3) $$b_{\mathrm{RP}}=\frac{1}{2}\sqrt{{\left(\|{\vec{d}}_{\mathrm{RP}}\|+\|{\vec{d}}_{\mathrm{RP}}-{\vec{y}}_{\mathrm{RP}}\|\right)}^{2}-{\left\|{\vec{y}}_{\mathrm{RP}}\right\|}^{2}}$$

(4) $${\vec{d}}_{R}={\vec{r}}_{R}-{\vec{r}}_{P}$$

(5) $${\vec{y}}_{\mathrm{RP}}=\left({\vec{v}}_{P}-{\vec{v}}_{R}\right)\times \Delta t$$

Additionally, the interaction force also depends on the angle of encounter, $\varphi_{\mathrm{RP}}$ . An angular-dependent prefactor, *$w\left(\varphi_{\mathrm{RP}}\left(t\right)\right)$ *, is introduced in Equations 6 and 7 and is multiplied by the dynamic repulsive force. This prefactor is derived to reflect the agent’s field of view, as agents are generally insensitive to other agents behind them. When the robot’s velocity coincides with the vector pointing from the robot to another agent, this prefactor remains at 1. However, when these two vectors are pointing in the opposite direction, the prefactor drops to $\lambda_{R}$ . $\lambda_{R}$ is a parameter ranging from 0 to 1 with increases in proportion to the intensity of interactions from behind.

(6) $$w\left(\varphi_{\mathrm{RP}}\left(t\right)\right)=\lambda_{R}+\left(1-\lambda_{R}\right)\times \frac{1+\cos\left(\varphi_{\mathrm{RP}}\right)}{2}$$

(7) $$\cos\left(\varphi_{\mathrm{RP}}\right)=\frac{{\vec{v}}_{R}}{\left\|{\vec{v}}_{R}\right\|}\times \frac{-{\vec{d}}_{\mathrm{RP}}}{\left\|{\vec{d}}_{\mathrm{RP}}\right\|}$$

The fourth term is the repulsive force from static obstacles in the environment. It is formulated in the same way as the forces induced by dynamic obstacles. However, the variables relevant to dynamic agents (i.e., humans in the environment) are now replaced by those relevant to static obstacles. The velocity of the static obstacles is set to zero in the simulation.

The simulated robot’s velocity and position are updated using Equations 8 and 9. Finally, the simulated velocity is capped at the maximum value of 1.44 m/s to reflect the highest speed the robot reached in the empirical experiments.

(8) $${\vec{v}}_{R}\left(t+1\right)={\vec{v}}_{R}\left(t\right)+{\vec{f}}_{R}\left(t\right)\times \Delta t$$

(9) $${\vec{r}}_{R}\left(t+1\right)={\vec{r}}_{R}\left(t\right)+{\vec{v}}_{R}\left(t+1\right)\times \Delta t$$

The robot is modeled as a MassMotion agent whose movement is entirely controlled by the modified SFM described in the section covering implementation of the robot model. The proposed algorithm is coded in C# using the MassMotion SDK. It is assumed that the robot has a cylindrical shape with a radius of 0.35 m, and the model is updated every 0.2 s. The cylindrical representation simplifies the mathematical modeling of HRI and helps to balance computational efficiency with realistic behavior simulation. This simplification is compensated by a rigorous calibration process to ensure a realistic reflection of the behavior of the robot.

## Robot Model Calibration and Validation

Calibration was performed to ensure that the proposed robot model behaved as realistically as possible. Implemented through a genetic algorithm (GA), a hybrid strategy combining empirical trajectory data with microscopic simulation data of the robot movement was applied for robot model calibration.

### Calibration Method

**Figure 6.**
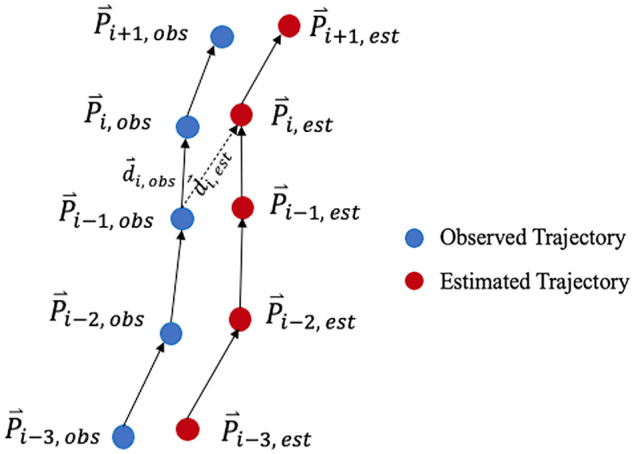
Illustration of observed and simulated trajectories.

The parameters to be calibrated were the 12 key parameters in the modified SFM. Each solution contains a set of social-force parameter values coded into binary strings of various lengths. When strung together, these parameters create a chromosome with a binary string length of 72. The first generation of the GA comprises a population of 30 random chromosomes, which represent potential solutions. In MassMotion, an agent (i.e., the virtual robot model) is moved according to the modified SFM with a set of parameters represented by a chromosome within the population. The operator and the pedestrian are moved according to the recorded trajectories in the experiments, and static obstacles are placed at the recorded locations. The model is run for each experimental scenario, and the resulting robot trajectories are compared against the recorded robot trajectories in that particular experiment using the relative distance error, $d_{\mathrm{err}}$ (*61*), as shown in Figure 6 and calculated as in Equations 10, 11, and 12. The relative distance errors of all data sets are then summed and refactored to be within the range of (0, 1) using Equation 13.

(10) $$\mathrm{relative}\;\mathrm{distance}\;\mathrm{error},d_{\mathrm{err}}=\frac{1}{m}\sum_{i=1}^{m}\left|\frac{d_{i}^{\mathrm{est}}-d_{i}^{\mathrm{obs}}}{d_{i}^{\mathrm{obs}}}\right|$$

(11) $$d_{i}^{\mathrm{est}}=\left|{\vec{r}}_{i}^{\mathrm{est}}-{\vec{r}}_{i-1}^{\mathrm{obs}}\right|$$

(12) $$d_{i}^{\mathrm{obs}}=\left|{\vec{r}}_{i}^{\mathrm{obs}}-{\vec{r}}_{i-1}^{\mathrm{obs}}\right|$$

(13) $$\mathrm{fitness},f=1-\frac{\sum_{j=1}^{n}d_{\mathrm{err}}}{10000}$$

where

${\vec{r}}_{i}^{\mathrm{obs}}$ is *i*th observed position vector;

${\vec{r}}_{i}^{\mathrm{est}}$ is *i*th estimated position vector;

*m* is number of positions in each data set; and

*n* is number of data sets being calibrated.

Out of the 130 data sets obtained during the experiments, two-thirds of the data sets from each subexperiment were randomly chosen for calibration, and the remaining were used for validation.

### Calibration and Validation Results

The 12 parameters obtained after the GA had iterated through 20 generations are given in Table 2. Then, to validate the proposed robot navigation algorithm, the modified SFM was applied to the remaining data sets with the calibrated parameters. For all experiments, the total relative distance error was 1,724.6 m for the calibration data set, with the fitness value being 0.83. For validation, the total relative distance error was 785.9 m, and the fitness value reached 0.92. After normalizing the total relative distance error by the number of data sets, the average relative distance errors for the calibration and validation sets were 19.8 and 18.3 m, respectively.

**Table 2.** Calibration Parameters from the Modified Social Force Model

Parameter	Unit	Value	Description
*s*	s	0.54	Relaxation time: time to match the desired velocity
$\lambda_{\alpha O}$	na	0.13	Visual field effect parameter (bot to operator)
$\lambda_{\alpha P}$	na	0.20	Visual field effect parameter (bot to pedestrians)
$A_{O}$	m/s^2^	1.67	Operator force strength: interaction strength for repulsive force from its operator
$B_{O}$	m	2.24	Operator force range: interaction range for repulsive force from its operator
$C_{O}$	m	2.62	Maximum operator range: the distance within which the robot reacts to its operator
$A_{P}$	m/s^2^	0.38	Neighbor force strength: interaction strength for repulsive force from surrounding pedestrians
$B_{P}$	m	4.75	Neighbor force range: interaction range for repulsive force from surrounding pedestrians
$C_{P}$	m	1.86	Maximum neighbor range: the distance within which the robot reacts to surrounding pedestrians
$A_{i}$	m/s^2^	0.14	Obstacle force strength: interaction strength for repulsive force from surrounding obstacles
$B_{i}$	m	0.62	Obstacle force range: interaction range for repulsive force from surrounding obstacles
$C_{i}$	m	2.27	Maximum obstacle range: the distance within which the robot reacts to surrounding obstacles

*Note:* na = not applicable.

**Figure 7.**
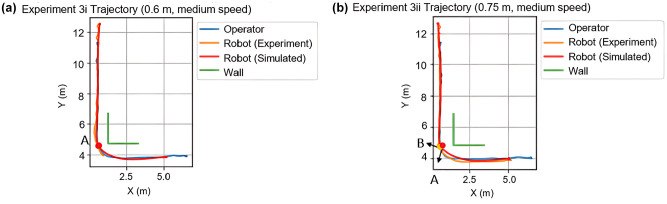
(*a*) Experiment 3i trajectory comparison, and (*b*) Experiment 3ii trajectory comparison.

**Figure 8.**
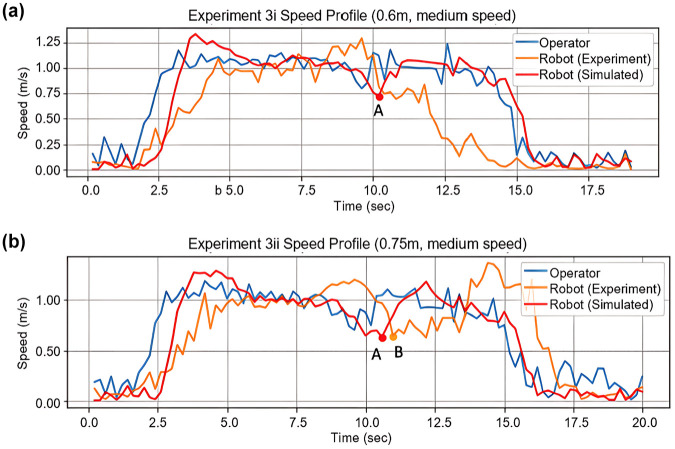
(*a*) Experiment 3i speed profile comparison, and (*b*) Experiment 3ii speed profile comparison.

The percentage of relative distance error from each experiment can be found in Table 3. The results suggest that the calibrated model produced a fit that was within 2.5% for 8 out of the 12 experiments. The relative distance error was within 2.5% for simple following and obstacle-avoidance scenarios. However, the model lacked accuracy when the robot needed to make sharp turns or when the operating space was extremely confined. Experiment 3 contributed the most error to both the calibration and the validation. When the operator’s intended walking speed was around 4 km/h and the distance from their route to the wall was 0.75 m, the simulated trajectory and speed profile were similar to the empirical observation, where the actual and simulated robot both reached their lowest speed approaching the corner at Points A and B (Figures 7*b*, 8*b*). However, in Experiment 3i, where the actual robot ceased to follow at the corner, the virtual robot continued to follow till the end of the route (Figures 7*a*, 8*a*). In both simulated cases, the virtual robot’s speed visibly dropped when it passed the corner, but the resultant force from the operator and the wall was unable to stop the virtual robot entirely in Experiment 3i. This could be because the simulated forces exerted on the robot were not as exhaustive as those in real life, which suggests possible improvements on the proposed algorithm.

**Table 3.** Percentage Relative Distance Error

Categories		Experiments		Percentage relative distance error		Percentage difference
				Calibration	Validation	
A	Robot+Operator	1	Straight line following test	2.42	2.40	−0.83
		2	Circular line following test	5.34	5.43	1.55
B	Robot+Operator+Static obstacles	3	Corner test	59.74	51.46	−13.87
		4	Static obstacle test (passing)	1.34	2.17	62.53
		5	Static obstacle test (avoidance)	1.67	1.68	0.42
		6	Obstacle map	1.69	1.70	0.59
C	Robot+Operator+Pedestrian	7	Pedestrian interference test (surpass)	1.77	1.85	4.69
		8	Pedestrian interference test (encounter)	1.73	1.71	−1.10
		9	“Lose target” test (crossing)	0.25	NA	NA
		10	“Lose target” test (takeover)	0.82	NA	NA
D	Robot+Operator+Static obstacles+Pedestrian	11	Static & dynamic obstacles test (surpass)	11.12	10.95	−1.59
		12	Static & dynamic obstacles test (encounter)	12.11	20.66	70.62

*Note:* NA = not available.

Except for Experiments 4 and 12, there seemed to be reasonably small differences between the validation and calibration results. The validation results aligned reasonably with the calibration results, indicating the model’s robustness in reproducing the robot’s observed behavior under normal working conditions. However, the results may suggest insufficient calibration for situations requiring more nuanced maneuvering. Since the parameters were optimized globally for all experiments at once, it was expected that the calibrated model would not fit individual experiments perfectly, especially for extreme cases. It is also important to note that this paper proposes a way to represent a sophisticated commercial robot using a relatively simple modified SFM. Although the most representative set of parameters was selected from the calibration process, the actual navigation algorithm could be different from the proposed one, and therefore differences were expected between the simulation and experimental results. Taking the above discussions into consideration, Section 6, evaluates the robot’s performance in a single corridor, with no sharp turns around corners, to ensure that the model was applied within its defined limitations.

## Evaluation of Robot Performance

An agent-based simulation model of an indoor pedestrian corridor was developed to assess the performance of the person-following robot under varying pedestrian crowding scenarios. The effects of average pedestrian density, directions of pedestrian flow, and corridor width were evaluated. This simulation model mainly serves as a practical application of the proposed modified SFM depicting the behavior of the robot. Such an application is useful to proactively evaluate the performance of the robot before it is introduced into a real pedestrian environment, such that it allows for more effective planning via feedback, which is the main objective of this analysis. Testing scenarios also help in identifying key aspects of the robot that may require modification to secure a more successful and wider application. This is crucial if the intended pedestrian environment is more complicated in relation to crowd behavior, geometric setup, or both. For example, the proposed model could be expanded to include a diverse range of pedestrian profiles, a bigger fleet of person-following robots, or a complex setup such as the layout of a floor in a shopping center or underground pedestrian network like PATH, which exists under Union Station in Downtown Toronto.

### Simulation Setup

To evaluate the performance of the robot in a dynamic pedestrian environment, an agent-based simulation model of a hypothetical 100-m indoor pedestrian corridor was developed in MassMotion (*45*). The agents included pedestrians, an operator, and a follower robot. The robot moved within the flow of pedestrians in response to the operator and in accordance with the modified SFM. Two portals were placed at both ends of the corridor, 2.5 m from the latter’s edge, to serve as origins and destinations for the agents. A journey was characterized by an origin and a destination.

The impacts of varying three parameters were evaluated: (1) pedestrian density, (2) directions of pedestrian flow with respect to the operator and the robot, and (3) corridor width. For each scenario, the trajectory of the robot was tracked throughout the simulation and recorded every 0.2 s, including its location in the corridor (X and Y coordinates) and instantaneous speed. A warmup period of 2.5 min was considered to ensure that the assumed pedestrian density was achieved before the operator and the robot were generated. The simulation period was 5 min. Each scenario was run 30 times with a different random seed per run.

For the analysis, records for the first and last 10-m sections from each portal were disregarded, to avoid boundary effects. Therefore, the effective portal-to-portal distance was 75 m. In all scenarios, it was assumed that the operator sought to maintain a speed of 1.44 m/s throughout its planned journey from one portal to another along the corridor, as this was the maximum speed that the robot could achieve.

### Description of Scenarios

Pedestrian density is the number of people occupying a unit area of space at a specific instant. Fruin extended the concept of level-of-service (LOS) analysis to pedestrian walkways (*62*). Six LOS rankings, A through F, are assigned to ranges of pedestrian density, such that LOS A is associated with low-density crowds and LOS F is associated with the highest-density crowds. We analyzed pedestrian densities ranging from LOS A to D. First the average pedestrian density was varied from zero to 0.75 pedestrians per square meter (ped/m^2^) at increments of 0.05 ped/m^2^, resulting in 16 scenarios. In these scenarios, the corridor width was fixed at 5 m and all pedestrians were assumed to flow opposite to the operator and the robot.

Low-density regimes comprised pedestrian densities less than 0.25 ped/m^2^ or a pedestrian flow rate less than 100 pedestrians per minute (ped/min); medium-density regimes comprised densities at least equal to 0.3 ped/m^2^ but fewer than 0.5 ped/m^2^ or pedestrian flow rates less than 200 ped/min; and lastly, high-density regimes comprised densities, at most, equal to 0.75 ped/m^2^ or pedestrian flow rates less than 300 ped/min. Densities greater than 0.75 ped/m^2^ represented the failing conditions of the proposed model setup: such a case testing would have been impossible. Two main occurrences were observed under the said regimes. In the first instance under LOS E, the robot was introduced into the simulation and was seen to hover around the origin portal until all generated pedestrians crossed over the destination portal and then the operator–robot duo starts its journey in a near empty corridor. In the second instance under LOS F, the robot would only hover near the origin portal and remain there till the end of the simulation period, indicating that it could not, because of its design constraints, operate in such situations. Therefore, the operational range of the pedestrian density for the proposed simulation setup was LOS A to D.

Second, five configurations were considered for the pedestrian flow directions: (1) 100% opposite to the operator and the robot, (2) bidirectional with 25% of pedestrians moving with the robot and 75% moving opposite to it, (3) bidirectional with 50% of pedestrians in each direction, (4) bidirectional with 75% of pedestrians moving with the robot and 25% moving opposite to it, and (5) 100% with the operator and the robot. In these five scenarios, three pedestrian density levels were evaluated, low (0.1 ped/m^2^), medium (0.4 ped/m^2^), and high (0.7 ped/m^2^), and the corridor width was fixed at 5 m. The total number of scenarios in this set was 15. Third, to test boundary effects, the corridor width was varied from 1 to 5 m at increments of 1 m, and then from 10 to 20 m at increments of 5 m, resulting in eight scenarios. In these scenarios, the pedestrian density was fixed at 0.25 ped/m^2^, which reflects a low-density regime without crowding. Like the first set of scenarios, the pedestrians flowed in the opposite direction to the operator and the robot.

We considered the robot’s mean speed and its variability, as well as journey distance and time, as performance measures. The mean speed was calculated as the average of the instantaneous speeds recorded every 0.2 s throughout the simulation run of a scenario. The journey distance was calculated as the summation of the Euclidean distances between the subsequent locations of the robot recorded along the corridor, noting that the robot could travel laterally to avoid pedestrians. The journey time was measured as the duration elapsed from the instant the robot traversed 10 m from its origin portal to the instant it reached a location that was 10 m from its destination portal.

### Impacts of Varying Pedestrian Density

**Figure 9.**
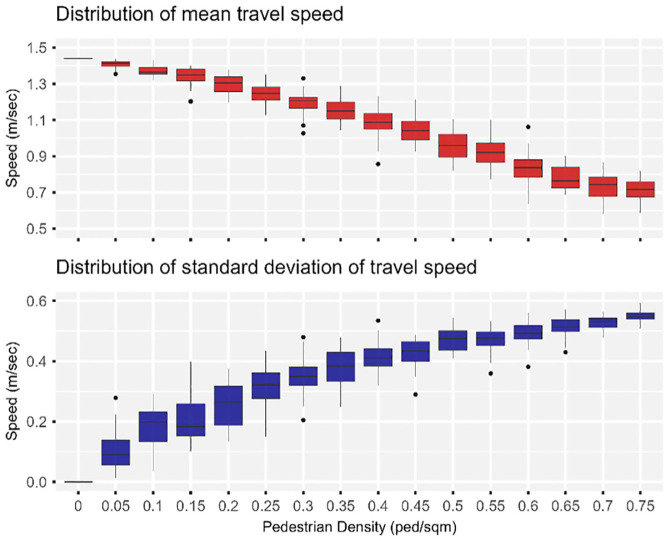
Effect of varying pedestrian density on the robot’s mean speed.

The average pedestrian density was varied from zero to 0.75 ped/ m^2^ at increments of 0.05 ped/m^2^. In other words, with a fixed corridor area of 500 m^2^, the total number of pedestrians generated per run was varied from zero to 1,500, at increments of 100. Figure 9 illustrates the distribution of the robot’s mean speed and corresponding standard deviation. As the pedestrian density was increased from zero to 0.75 ped/m^2^, the average of the robot’s mean speed across the runs of a scenario dropped from a constant of 1.44 to 0.72 m/s, 95% CI [0.69, 0.74]. Between scenarios, the variability of the mean speed increased as the corridor became more crowded. This means that the standard deviation of the robot’s mean speed increased from zero, when there were no pedestrians, to an average of 0.55 m/s, 95% CI [0.54, 0.56], when the pedestrian density was increased to 0.75 ped/m^2^. However, within the scenarios, the variance of the standard deviation was reduced as the pedestrian density was increased, as shown in Figure 9.

**Figure 10.**
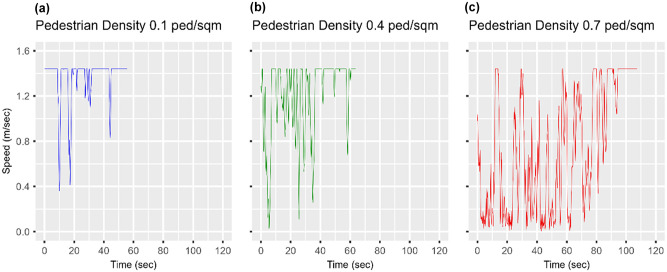
Effect of varying pedestrian density on the robot’s speed profile: (*a*) 0.1 ped/m^2^, (*b*) 0.4 ped/m^2^, and (*c*) 0.7 ped/m^2^.

**Figure 11.**
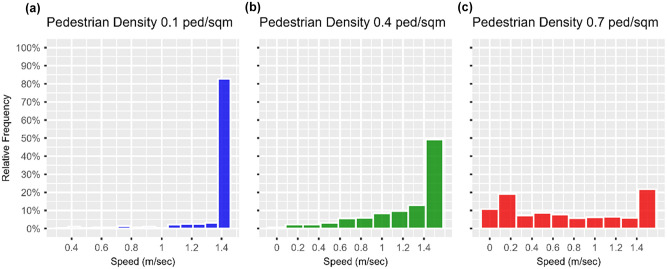
Effect of varying pedestrian density on the distribution of robot’s speed: (*a*) 0.1 ped/m^2^, (*b*) 0.4 ped/m^2^, and (*c*) 0.7 ped/m^2^.

The intra-scenario variation of the robot’s speed were given a closer look. The scenarios were grouped into three levels based on their respective pedestrian density. Low-density scenarios comprised Scenarios 1 through 5, in which the pedestrian density varied from zero to 0.20 ped/m^2^. Medium-density scenarios comprises Scenarios 6 through 10, in which the pedestrian density varied from 0.25 to 0.45 ped/m^2^. High-density scenarios comprised Scenarios 11 through 16, in which the pedestrian density was varied from 0.50 to 0.75 ped/m^2^. A single scenario was selected from each level, specifically, Scenarios 3, 9, and 15, in which the pedestrian density was increased from 0.10 to 0.70 ped/m^2^ at increments of 0.30 ped/m^2^. Figure 10 illustrates the variation of the robot’s speed versus time elapsed for a randomly selected run for Scenarios 3, 9, and 15. Figure 11 displays the relative frequency histogram plots of the robot’s speed for corresponding Scenarios 3, 9, and 15.

In Scenario 3, in which the pedestrian density was low (0.10 ped/m^2^), the mean speed of the robot was 1.37 m/s, 95% CI [1.35, 1.39], and more than 90% of the recorded speeds ranged between 1.22 and 1.44 m/s. About 59% of the journey was traveled at a speed equal to 1.44 m/s, which was the maximum that the robot could achieve. When the pedestrian density was increased to 0.40 ped/m^2^ in Scenario 9, the robot’s mean speed dropped to 1.09 m/s, 95% CI [1.05, 1.14]. About 90% of the recorded speeds ranged between 0.46 and 1.44 m/s, indicating an increase in the variability of the robot’s speed compared with Scenario 3 in which the pedestrian density was lower. Also, the portion of the robot’s journey traveled at 1.44 m/s dropped to 23%. Lastly, when the pedestrian density was increased to 0.70 ped/m^2^, the mean speed dropped to 0.66 m/s, 95% CI [0.61, 0.70], with about 20% of recorded speeds dropping below 0.12 m/s and only 14% of robot’s journey traveled at 1.44 m/s.

The effects of varying the pedestrian density on the robot’s journey distance and time were also evaluated. The robot’s journey distance increased as the corridor became more crowded with pedestrians. In addition to the effective portal-to-portal distance (75 m) that the robot traveled to reach its destination at the west portal, it was forced to also deviate laterally to avoid bumping into pedestrians. A noticeable increase in the journey distance was observed when the pedestrian density exceeded 0.40 ped/m^2^. At a pedestrian density of 0.75 ped/m^2^, the robot approximately traveled an additional 3.4 m, 95% CI [2.9, 3.9] (i.e., about 4.5% of the base case journey length of 75 m). Additionally, as the robot’s mean speed was reduced and it was forced to travel additional distance laterally, the journey time increased to about 112 s, 95% CI [108, 116], which was approximately twice as long as the free-flow travel time of 53 s when there were no pedestrians. For each additional meter in lateral deviation, the journey time increased by approximately 21 s, on average.

### Impacts of Varying Pedestrian Flow Directions

**Figure 12.**
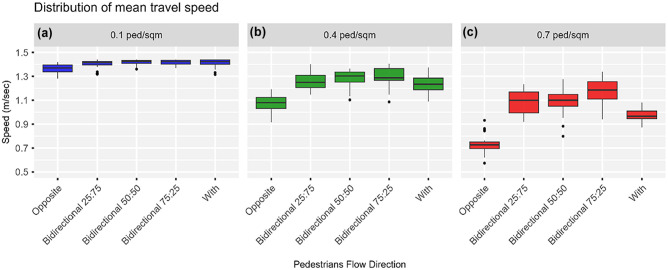
Effect of varying pedestrian flow directions on the robot’s mean speed: (*a*) 0.1 ped/m^2^, (*b*) 0.4 ped/m^2^, and (*c*) 0.7 ped/m^2^.

Figure 12 depicts the distribution of the robot’s mean speed under different pedestrian flow configurations and pedestrian densities. Generally, the robot’s mean speed improved as the fraction of pedestrians walking in the same direction increased from 0 to 100% at increments of 25%. Nonetheless, similar to the results discussed in the previous section, the increase in pedestrian density had an adverse effect on the robot’s mean speed regardless of the configuration of the pedestrian flow. Under low-density regimes (i.e., average pedestrian density of 0.10 ped/m^2^), the speed slightly increased by 3% to 4% from 1.36 m/s, 95% CI [1.35, 1.37] regardless of the fraction of pedestrians walking in the same direction as the operator and the robot. This means that in the least crowded areas, the configuration of pedestrian flows had a minor impact on the performance of the robot.

When the average pedestrian density was increased to 0.40 ped/m^2^, the improvement in the robot’s mean speed increased. Having only 25% of the pedestrians walk in the same direction as the operator and the robot, resulted in about an 18% increase in the robot’s mean speed from 1.07 m/s, 95% CI [1.04, 1.10], when all pedestrians were set to walk opposite to the operator and robot. However, further increasing the fraction of pedestrians moving with the operator and the robot to 50% and 75%, only improved the robot’s mean speed by an additional 2% and 4%, respectively. The increase in the robot’s mean speed dropped to about 15% when all pedestrians were set to move with the operator and the robot under medium-density regimes. This could be the result of the operator and the robot being delayed behind slower-moving pedestrians.

The robot’s mean speed dropped to 0.73 m/s, 95% CI [0.70, 0.76] on increasing the average pedestrian density to 0.70 ped/m^2^ when all pedestrians were set to walk in the direction opposite to the operator and the robot. Under such a high-density regime, having more pedestrians walking in the same direction as the operator and the robot resulted in considerable improvement in the robot’s performance, more so than under medium-density regimes. When 25% or 50% of the pedestrians moved with the robot, the robot’s mean speed increased by up to 49%. An additional 13% increase in the mean speed was observed when 75% of pedestrians walked in the same direction as the robot. Nonetheless, like medium-density regimes, when all pedestrians were set to move with the robot, the robot’s mean speed improved by 33%, and this was in part the result of being hampered by slow pedestrians.

Whereas changing pedestrian flow directions with respect to the operator and the robot had little to no impact on the robot’s journey under low- to medium-density regimes, a more significant improvement was noted for high-density regimes. When 25% of the pedestrians were set to move in the same direction as the robot, the robot conducted less lateral deviation such that the journey length was almost equal to the effective portal-to-portal distance of 75 m. More noticeable was the improvement in the journey time, which was reduced from 109 to 71 s; this was mainly attributed to the improvement in the robot’s average speed. Further increasing the fraction of pedestrians moving with the robot to 50%, 75%, and 100% resulted in slighter improvements in the journey time.

### Impacts of Varying Corridor Width

**Figure 13.**
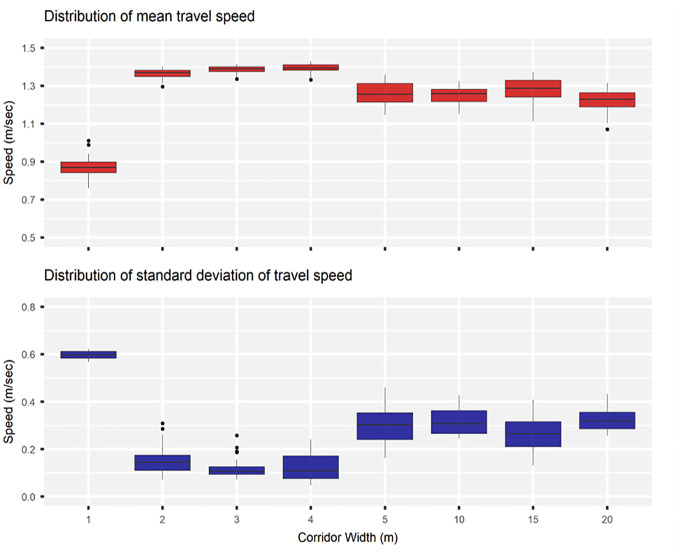
Effect of varying corridor width on the robot’s mean speed.

Boundary effects on the robot’s performance were evaluated by varying the corridor width. First, the width was increased from 1 to 5 m at increments of 1 m. Second, it was increased from 10 to 20 m at increments of 5 m. A medium-density regime was adopted, such that the average pedestrian density was fixed at 0.25 ped/m^2^ for the considered widths. Also, all pedestrians were set to move opposite to the operator and the robot. A 1-m wide corridor obstructed the mobility of the operator and the robot, and this resulted in the robot traveling at 40% lower than its maximum speed of 1.44 m/s. Widening the corridor to 2 m improved the robot’s mean speed by 56% from 0.87 m/s; however, further widening it to 3 and 4 m, resulted in little additional improvement, increasing the robot’s mean speed by about 60%. Further increasing the corridor width to 5, 10, 15, or 20 m did not result in additional improvement in the robot’s mean speed; on the contrary, the mean speed dropped approximately 9% to an average of 1.26 m/s. Figure 13 illustrates the variation in the distribution of the robot’s mean speed with respect to changing the corridor width.

### Summary of Key Findings

First, the pedestrian density was varied from zero to 0.75 ped/m^2^ at increments of 0.05 ped/m^2^, while the corridor width was fixed at 5 m and all pedestrians were set to walk in the direction opposite to the operator and the robot. It was found that as the corridor became more crowded, the robot’s mean speed dropped gradually until it reached a minimum of 0.72 m/s, which was two times less than its maximum speed. As the pedestrian density increased, shorter portions of the robot’s journey were traveled at 1.44 m/s, such that speeds as low as 0.12 m/s were recorded for 20% of its journey when the pedestrian density was at a high of 0.70 ped/m^2^. Lower speeds and frequent lateral deviation lengthened the robot’s journey to twice the free-flow travel time of 53 s.

Second, five configurations of pedestrian flow directions were considered. The first scenario assumed that all pedestrians moved opposite to the operator and the robot, whereas the last scenario assumed that all agents moved in the same direction. Bidirectional flow was also considered, whereby the fraction of pedestrians walking in the same direction as the robot was 25%, 50%, and 75%. Although the robot’s speed improved when more pedestrians moved with the robot, this improvement was less significant when all the pedestrians were moving in the same direction as the robot, since the operator and the robot could find themselves stuck behind slower-moving pedestrians. Nonetheless, bidirectional flow was more convenient for the robot, especially under medium- and high-density regimes. Improvements of up to 62% in the travel speed of the robot were observed when 75% of the pedestrians were set to move in the same direction as the robot when the pedestrian density was 0.70 ped/m^2^.

Lastly, boundary effects of the corridor were also evaluated, such that the performance of the robot was assessed in response to varying the corridor width. The width was increased from 1 to 5 m at increments of 1 m, and from 10 to 20 m at increments of 5 m. In these scenarios, a medium-density regime (0.25 ped/m^2^) was assumed, and all pedestrians were set to move opposite to the operator and the robot. It was found that widening the corridor by only 1 m significantly improved the robot’s speed; however, each additional meter in width resulted in smaller speed improvements.

## Conclusions and Future Work

This paper presents a simulation approach to evaluating the performance of a person-following delivery robot in dynamic pedestrian environments. Since it is a commercial robot designed by a private company, it is a black box system whose operating characteristics can only be gained through empirical observations. As a result, rigorous laboratory experiments were conducted to observe how the robot follows a person and how it behaves when confronting various static and dynamic obstacles in its working environment.

The experiments showed that the robot was able to maneuver around static and dynamic obstacles and would always maintain sufficient distance from them, but it might stop and require human intervention in rare cases where two obstacles were both in its moving direction and were blocking escape routes. It was also found that the robot was more sensitive to the obstacles ahead of it, in the moving direction, than on its sides, meaning that it would keep a longer distance to the obstacles in front of it than to those next to it. The robot was able to differentiate between its operator and a pedestrian when the pedestrian crossed between the robot and the operator, and it would always follow the operator. This feature is crucial if it were to be deployed in a pedestrian-dense area.

In general, the robot could follow the operator with more precision if the operator walked at a slow to medium walking speed, especially when the operator was making sharp turns, as the robot’s movement could be significantly hindered by its kinematic system. Therefore, it is advised that the courier maintains a low to medium walking speed when making turns and takes routes farther from obstructions to ensure smooth operation in real-life practices.

Based on the experimental observations, a modified SFM was developed to represent a virtual person-following robot in the pedestrian simulation software, MassMotion. Calibration of the model was performed against the experimental data by applying a GA, and the model was validated using hold-out data. It was found that the calibration and validation results were mostly consistent in the percentage of relative distance error each experiment contributed to the overall error. For most experiments, the proposed algorithm replicated the observed behavior with minor discrepancies. However, the laboratory testing could only be completed in low-density pedestrian conditions, and while it was possible to extrapolate the results for medium- to high-density crowding scenarios, this extrapolation adds some uncertainty to the validity of the outcomes. The algorithm also did not perform well in the circular line following test, corner test, and static & dynamic obstacles test (encounter). One of the reasons this happened is that the modified SFM uses the direction-following approach, which often leads to the follower cutting corners and not following the leader’s exact footsteps. Additionally, the parameters of the modified SFM were optimized globally for the different experiments, which means that the model may not be a perfect fit for the individual experiments. The other explanation involves limitations of the research, which are discussed below.

As mentioned earlier, one of the biggest challenges of this research was replicating a complex robotics system with a single social-force-based model. Especially when a system contains proprietary technologies, it is only possible to reverse-engineer a robot’s behavior with the limited data available, which here were the time-stamped location data of the robot and the obstacles it interacted with during the experiments. The I/O sensor data and kinematic architecture of the robot were not considered in this study. As a result, the simulated robot manifested some unrealistic behavior, such as moving along a route with a turning radius that was smaller than the minimum threshold of 1.2 m. The simulation could not realistically reflect the technological limitations under extreme cases, such as going around a corner too sharply. It is assumed that in real-life applications, the operators would account for the robot deficiencies under extreme cases. The inclusion of vehicle kinematics and the assessment of the robot’s performance in more complicated situations can be explored in future studies.

An agent-based simulation was also conducted to evaluate the performance of a person-following robot under varying crowding scenarios. The simulation environment consisted of a hypothetical 100-m long pedestrian corridor. The robot was set to follow an operator according to a modified SFM, sharing the corridor with pedestrians moving per the SFM developed by Helbing and Johansson (*38*). The performance of the robot was assessed for its mean travel speed, as well as for the average journey length and time. The journey length comprised the effective portal-to-portal distance of 75 m plus any additional lateral deviation conducted by the robot to avoid pedestrians. Overall, it was found that adopting low- to medium-density regimes and bidirectional pedestrian flow along indoor corridors comprised an optimal environment for a person-following robot to operate efficiently. Beyond eliminating boundary effects, varying the corridor width had a minor impact on the performance of the robot. The calibrated model was applied and evaluated within the confines of a straight corridor; that the robot does not perform well under extreme conditions is irrelevant to the evaluation results.

Potential research arising from this study includes refining the proposed force-based algorithm, and adding vehicle kinematics into the simulation model to obtain a higher level of accuracy when simulating robot behavior in difficult situations. Then, it would be useful to assess the performance of the person-following robot in more complex pedestrian environments, compare the simulated behavior of a person-following robot to the observed behavior of a similar robot operating in real life, and explore the potential of fully ADRs in similar pedestrian environments.

This paper presents a model that reasonably reproduced the behavior of a person-following delivery robot under various working conditions, which could be readily integrated into a pedestrian simulation environment. More importantly, this research approach is versatile and could be applied to other commercial autonomous robots in the market. It is believed that the proposed simulation study could facilitate the regulatory acceptance of delivery robots in public spaces by providing objective, data-driven insights into their performance and impact. Detailed metrics, such as efficiency, could be derived from simulations that cover a wide range of scenarios, with minimal risk. Simulations are also more cost-effective and scalable than extensive field testing. The results could guide recommendations for deployment conditions, ensuring that robots are used in ways that comply with public safety expectations.
